# Draft Genome Sequence of Pseudarthrobacter sp. Strain AG30, Isolated from a Gold and Copper Mine in China

**DOI:** 10.1128/MRA.01329-18

**Published:** 2018-11-01

**Authors:** Ibtissem Ben Fekih, Yuanjing Ma, Martin Herzberg, Chengkang Zhang, Yuan Ping Li, Sohaib H. Mazhar, Suleiman Kehinde Bello, Nan Yang, Junming Su, Junqiang Xu, Ruirui Zhang, Renwei Feng, Zhengxian Chen, Christopher Rensing

**Affiliations:** a Institute of Environmental Microbiology, College of Resources and Environment, Fujian Agriculture and Forestry University, Fuzhou, China; b College of Life Science, Fujian Agriculture and Forestry University, Fuzhou, China; c Molecular Microbiology, Institute for Biology/Microbiology, Martin-Luther-University Halle-Wittenberg, Halle (Saale), Germany; d Zijin Mining Group Co., Ltd., Shanghang, Fujian, China; Indiana University Bloomington

## Abstract

Here, we report the features and draft genome sequence of Pseudarthrobacter sp. strain AG30, isolated from the Zijin gold and copper mine in China. The genome size of Pseudarthrobacter sp. AG30 was 4,618,494 bp, with a G+C content of 66.2%. Interesting genes and operons putatively conferring resistance to copper and arsenic were identified.

## Abstract (precis)

Here, we report the features and draft genome sequence of Pseudarthrobacter sp. strain AG30, isolated from the Zijin gold and copper mine in China.

## ANNOUNCEMENT

Pseudarthrobacter sp. strain AG30 was isolated from soil of a gold-copper mine at Zijin Mountain, Fujian, China. The high-quality draft genome sequence of this bacterium was analyzed to investigate a genomic island and associated operons allowing the adaptation of this strain to an environment containing high concentrations of heavy metals. The bacterium was isolated by inoculating the collected soil samples on Reasoner's 2A (R2A) agar medium and selecting for growth on levels of heavy metals. A single colony of the isolated strain AG30 was grown aerobically in 40 ml of R2A broth culture incubated at 30°C with shaking at 300 rpm. Total genomic DNA was extracted using the TIANamp bacteria DNA isolation kit as described by the manufacturer (TianGen Biotech, Beijing Co., Ltd.). Draft genome sequencing was performed using an Illumina HiSeq X Ten sequencer at Vazyme Biotech Co., Ltd. (Nanjing, China). The library was prepared using the Illumina V3 VAHTS universal DNA library prep kit according to the VAHTS universal DNA sample preparation protocol (Illumina) with a paired-end sequencing strategy (300-bp insert size). The 14,056,567 raw Illumina reads with 242× coverage were quality filtered, trimmed, and assembled *de novo* with default settings using CLC Genomic Workbench 11.0.1.0 (Qiagen, Hilden, Germany).

Functional annotation of predicted genes was obtained using the Rapid Annotations using Subsystems Technology (RAST) server (1) and the NCBI Prokaryotic Genome Annotation Pipeline (PGAP) (2). The analyses of the Pseudarthrobacter sp. AG30 genome revealed a genome size of 4,618,494 bp assembled into 90 scaffolds, with a GC content of 66.2% and an *N*_50_ value of 171,675 bp. As predicted by PGAP, Pseudarthrobacter sp. strain AG30 contained 4,288 predicted genes with 4,225 protein coding sequences, including 129 pseudo genes. In addition, 63 RNA genes were identified, including 10 rRNAs (5 5S RNAs, 2 16S RNAs, and 3 23S RNAs), 50 tRNAs, 2 noncoding RNAs (ncRNAs; RNase P RNA component class A and signal recognition particle small RNA [sRNA]), and 1 transfer-messenger RNA (tmRNA) gene. Based on RAST analysis, 410 subsystems were represented within the chromosome, representing 43% of the assigned sequence. Numerous genes were reported from Pseudarthrobacter sp. AG30 that are putatively involved in heavy metal tolerance. Interesting features of this metal resistome included two *ars* clusters containing *arsR*, encoding a putative arsenite-responsive repressor containing a helix-turn-helix motif; many *arsC* genes encoding arsenate reductases (3); and a gene encoding an aquaglyceroporin-like channel (AqpS) (4). In total, 5 hypothetical genes encoding different arsenate reductases were found in both operons combined. Both operons contained a gene encoding a flavin adenine dinucleotide (FAD)-dependent oxidoreductase, and one operon possessed *arsT*, encoding a thioredoxin reductase (5). Moreover, both *ars* operons contained genes encoding a putative *N*-acetyltransferase resembling ArsN (6).

In addition, two putative operons were reported, each encoding CsoR, CopZ, and CopA, with CsoR being the copper-responsive regulator (7), CopZ a copper chaperone, and CopA a Cu(I)-translocating P_IB-1_-type ATPase (8). Adjacent to one of the *cpoR-copZA* operons, there appears to be another copper resistance determinant-containing gene encoding a multicopper oxidase, a prolipoprotein diacylglyceryl transferase, and CopD, which is probably involved in copper uptake (9).

### Data availability.

This whole-genome shotgun (WGS) project has been deposited at DDBJ/EMBL/GenBank under the accession number QEHL00000000 (version QEHL01000000). The original sequence data can be found at the NCBI Sequence Read Archive under the SRA accession number SRP160413.
